# Surface Texture, Wettability and Tribological Behavior of Titanium Nitride-Based Coatings Deposited on Ti6Al4V Titanium Alloy

**DOI:** 10.3390/ma18215010

**Published:** 2025-11-03

**Authors:** Katarzyna Piotrowska, Monika Madej

**Affiliations:** Faculty of Mechatronics and Mechanical Engineering, Kielce University of Technology, al. Tysiąclecia Państwa Polskiego 7, 25-314 Kielce, Poland; mmadej@tu.kielce.pl

**Keywords:** TiCN and TiN:Ag coatings, surface texture, wettability, friction

## Abstract

This study presents an analysis of two titanium nitride-based coatings, TiCN and TiN:Ag, deposited on Ti6Al4V alloy by physical vapour deposition (PVD). The investigation focused on the characterisation of surface geometric structure and wettability, tribological parameters, and osseointegration. The purpose of the study was to obtain a better understanding of the interactions between the implant surface and the surrounding tissues and body fluids, which are essential for ensuring long-term durability. The results revealed significant differences in surface stereometric parameters between the coatings, with TiN:Ag exhibiting higher roughness values. These variations were reflected in the wettability tests, where the coating with a more developed surface topography (TiN:Ag) demonstrated contact angle values approximately 15% higher than those of TiCN. In contrast, tribological tests indicated superior performance of the TiCN coating, which exhibited lower coefficients of friction in artificial saliva at pH 5.8 and 6.8—reduced by 20% and 36%, respectively, compared with TiN:Ag. The findings confirmed that surface topography exerts a decisive influence on both wettability and tribological behaviour of the coatings, aspects that must be considered in their design for implantological applications.

## 1. Introduction

The dynamic development of dental implantology has resulted in an increasing demand for materials that ensure both functionality and long-term durability in the oral environment, which is characterised by variable pH, the presence of enzymes, proteins and microorganisms, as well as cyclic mechanical loading. Frequent implant failures associated with wear, loosening, instability or infection [1,2] have prompted the development of novel material solutions aimed at improving implant longevity and reliability through the enhancement of surface tribological and mechanical properties. According to literature data [3], the pH of saliva usually ranges between 6 and 7; however, it may decrease following the consumption of acidic foods or beverages, leading to the degradation of titanium surfaces, breakdown of the passive oxide layer and accelerated corrosion, which consequently promotes higher friction and wear. At the same time, salivary proteins such as mucin and albumin, as well as lipids and glycoproteins, may provide a protective effect by partially mitigating these processes, which underlines the significance of the chemical and biological environment in implant durability.

Previous studies have reported on functional coatings designed to reduce wear and to improve the resistance of implant surfaces to mechanical and biological factors. Ceramic coatings based on titanium carbonitride (TiCN) doped with silver (Ag) demonstrated a substantial reduction in friction coefficient and material loss under lubrication with simulated body fluids (HBSS) and HBSS supplemented with bovine serum albumin, with the effect being particularly pronounced on hydrophobic surfaces [4]. Similarly, Ti–C–N coatings deposited by the PVD cathodic arc process improved the wear resistance and reduced the friction coefficient of Ti6Al4V alloy [5]. In addition, TiCN coatings obtained by PVD exhibited a beneficial influence on tribological performance, reducing both the coefficient of friction and volumetric wear when tested under lubrication with artificial saliva [6].

Parallel studies have been conducted on carbide and metallic coatings such as diamond-like carbon (DLC), graphite-like carbon (GLC), TiN and Ta. These analyses included the evaluation of hardness, elastic modulus, hardness-to-modulus ratio and tribological performance in protein-based solutions simulating synovial fluid (diluted bovine serum). The tantalum coating exhibited the lowest wear coefficient under high contact loads, whereas DLC and TiN coatings were limited by internal stresses, susceptibility to delamination and reduced resistance to cyclic loading. The results of these studies indicate that the effectiveness of coatings depends not only on their chemical composition but also on deposition parameters, adhesion to the substrate, coating uniformity and their synergistic interaction with body fluids and adsorbed proteins [7].

The current challenges in dental implantology, including variable pH, the presence of enzymes, proteins and microorganisms, as well as cyclic mechanical loading in the oral cavity, necessitate the development of new materials resistant to corrosion, wear and infection [8,9,10,11]. Research on ceramic coatings such as silver-doped TiCN and the evaluation of their tribological and mechanical properties represents a promising direction for the development of durable and reliable dental implants. Despite extensive studies, there remains a lack of data on the behaviour of these coatings under conditions simulating the oral environment, particularly in the presence of artificial saliva at pH 5.8 and 6.8, corresponding to physiological fluctuations of oral pH. Assessment of tribological performance under such conditions is crucial for dental implantology, as it enables a more realistic simulation of in vivo implant conditions and a more accurate evaluation of their durability and wear resistance. The present study addresses this research gap and aims to optimise the performance of TiCN and TiN:Ag coatings for dental applications, thereby enhancing their clinical potential and safety.

## 2. Materials and Methods

Two titanium nitride-based coatings, TiCN and TiN:Ag (Table 1), were deposited on a Ti6Al4V titanium alloy substrate, which is widely used in implantology owing to its favourable strength-to-weight ratio and high biocompatibility [6,12,13,14,15,16,17]. The coatings were produced using the physical vapour deposition (PVD) technique, which enables control of the chemical composition and layer thickness while ensuring good adhesion to the substrate. To guarantee process repeatability and coating quality, the deposition was carried out under controlled cleanroom conditions by an external company specialising in PVD technologies. Information regarding the type of PVD process and deposition temperature was provided; however, detailed technological parameters are proprietary to the manufacturer and were not disclosed. In the case of the TiN:Ag coating, silver was introduced into the reactive atmosphere as a dopant, imparting antibacterial properties to the deposited layer. The Ti6Al4V alloy without coating was not tested in the experimental programme, as its tribological properties have been extensively reported in the literature [6,18,19,20], where it was shown to exhibit high wear under both dry friction and lubrication in fluids simulating body conditions.

**Figure 1:**
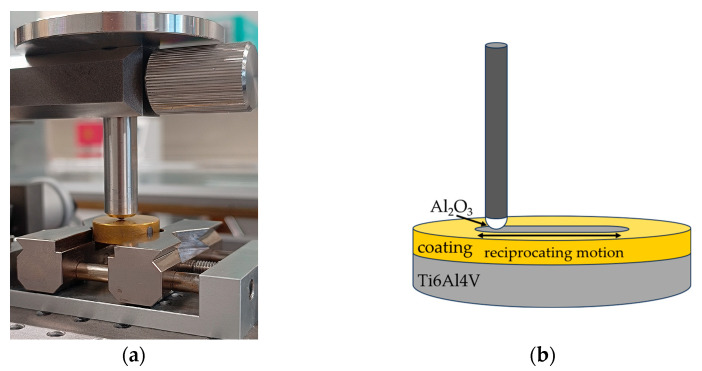
Friction pair: photography (**a**), diagram (**b**).

In the present study, the surface texture (Section 3.1) and wettability (Section 3.2) were analysed, as these parameters exert a considerable influence on tribological behaviour (Section 3.3). The evaluation of these features allows a better understanding of the interaction between the implant surface and the surrounding tissues and body fluids, which is crucial for osseointegration and, consequently, for the functional durability of implants [21,22]. The detailed experimental methodology is summarised in Table 2, and an example of the tribological test configuration is shown in Figure 1. After the completion of tribological tests, the wear tracks were examined microscopically (Section 3.3).

## 3. Results

### 3.1. Microstructure and Surface Texture Results

**Figure 2:**
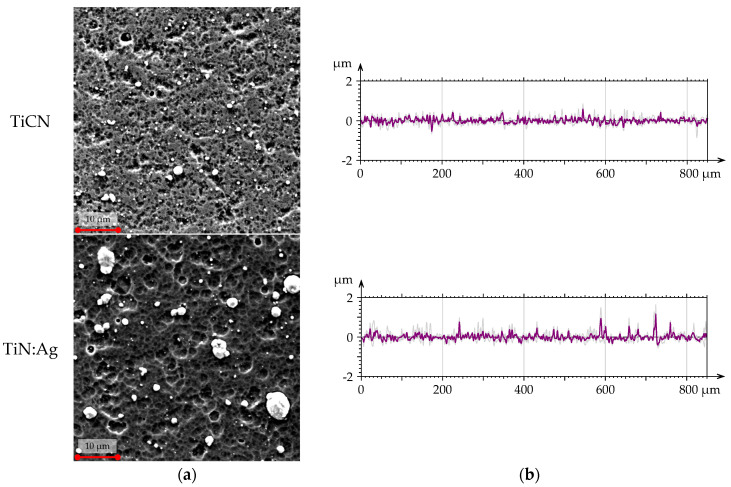
Surface texture: SED images (**a**) and primary profiles on cross-section (**b**) of TiCN and TiN:Ag coatings.

**Figure 3:**
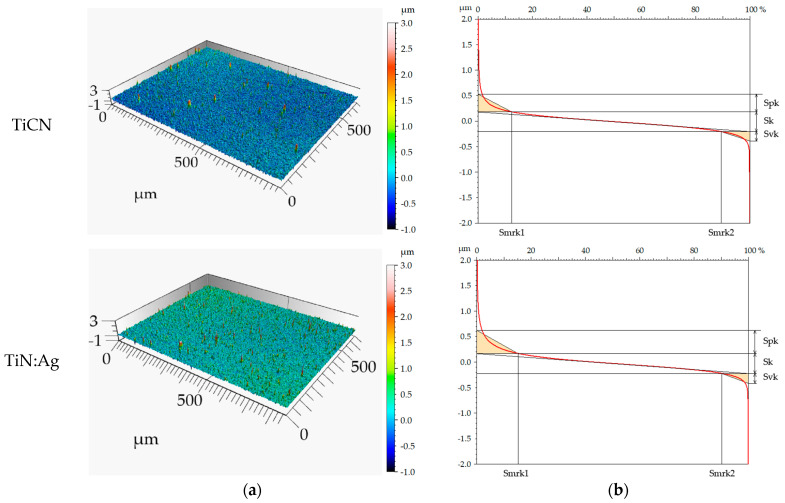
Three-dimensional axonometric images (**a**) and material contribution curves (**b**).

The geometric structure of the surface was analysed using scanning electron microscopy (SEM) (Figure 2) and confocal microscopy (Figure 3). The confocal microscope provided three-dimensional axonometric images, material contribution (Abbott–Firestone) curves, averaged primary profiles, and amplitude parameters (Table 3). The material contribution curves and the parameters extracted from them—namely the reduced peak height (Spk), reduced valley depth (Svk), and core roughness depth (Sk)—were used to further characterise the functional surface features (Table 4). All measurements were performed in accordance with the relevant standard [23].

Based on the analysis of SEM images, axonometric views, surface profiles, and roughness parameters, the TiN:Ag coating exhibited a more developed surface topography compared with the TiCN coating. The arithmetic mean height (Sa) for TiCN was 0.1 μm, whereas for TiN:Ag it was approximately twice as high. A similar trend was observed for the remaining height parameters, including the root mean square height (Sq), maximum peak height (Sp), and maximum valley depth (Sv), indicating the presence of more numerous and diverse surface irregularities in the silver-doped coating. These differences were also reflected in the statistical parameters—skewness (Ssk) and kurtosis (Sku). In both coatings, Ssk values were positive and Sku exceeded 3. This combination of parameters indicates the presence of deep valleys and sharp peaks with steep slopes, confirming a more irregular surface topography for the TiN:Ag coating.

The surface topography analysis of TiCN and TiN:Ag coatings included both height parameters and the material distribution characteristics described by the Abbott–Firestone curve. This curve illustrates the percentage contribution of individual height levels within the surface profile, enabling assessment of the relative proportions of peaks, core region, and valleys. Parameters derived from this curve, including the core roughness depth (Sk), reduced peak height (Spk), and reduced valley depth (Svk), provide information on the load-bearing capacity and lubricant retention capability of the surface, allowing a preliminary estimation of its tribological behaviour.

The results demonstrated that the TiN:Ag coating possessed higher roughness and a more developed geometric structure compared with the TiCN coating. This was confirmed by the higher values of amplitude parameters such as Sa, Sq, Sp, and Sv, as well as by positive skewness (Ssk) and kurtosis (Sku) values exceeding 3, which indicate the presence of sharp peaks and deep valleys. The parameters obtained from the material ratio curve corroborated these observations. The TiN:Ag coating exhibited higher Spk and Sk values, indicating the occurrence of numerous prominent peaks that may be gradually worn during the running-in phase before a stable contact surface is formed. Furthermore, a higher Svk value was observed for TiN:Ag, suggesting an enhanced ability to retain lubricants, which could improve its tribological performance under long-term operating conditions [24,25,26].

### 3.2. Contact Angle

**Figure 4:**
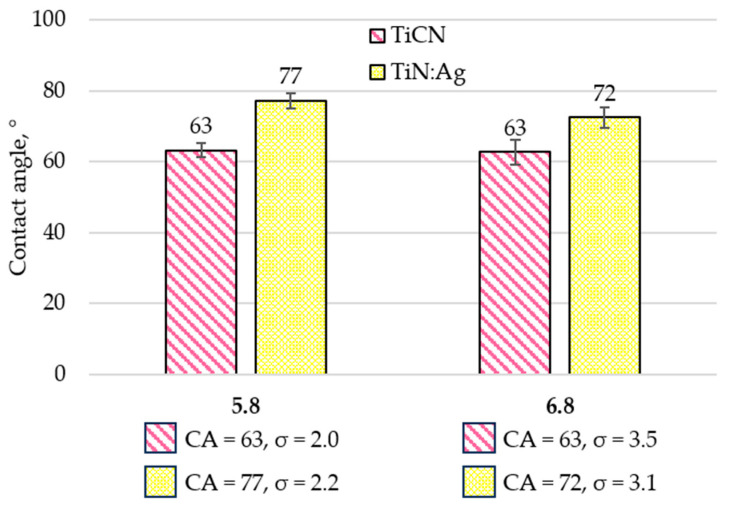
Average contact angle with artificial saliva at pH 5.8 and 6.8.

**Figure 5:**
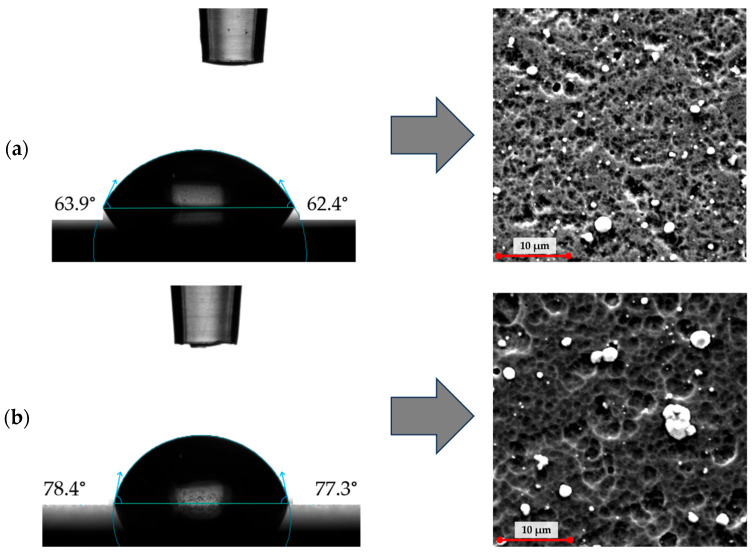
Representative contact angle images (artificial saliva, pH 5.8) and corresponding SEM micrographs of TiCN (**a**) and TiN:Ag (**b**) coatings.

The wettability of the coating surfaces was assessed by measuring the contact angle (CA), defined as the angle formed between the solid surface and the tangent to a liquid droplet at the point of contact between the solid, liquid, and gaseous phases. Lower contact angle values correspond to higher surface wettability [27,28,29]. The measurements were performed using the sessile drop method. A 5 µL droplet of artificial saliva solution with pH values of 5.8 and 6.8 was deposited on the surface of each coating. The tests were carried out under controlled laboratory conditions (temperature 23 ± 1 °C, relative humidity 55 ± 5%). For each coating, ten independent measurements were conducted, and the average contact angle values were calculated (Figure 4 and Figure 5).

Wettability tests performed using artificial saliva solutions at pH 5.8 and 6.8 revealed distinct differences in surface behaviour between the analysed coatings. For artificial saliva at pH 5.8, the contact angle measured for the TiCN coating was 63°, while for the TiN:Ag coating it reached 77°. At pH 6.8, the contact angle for TiCN remained at 63°, whereas TiN:Ag exhibited a slightly lower value of 72°. These results indicate a more hydrophilic character of the TiCN surface compared with the silver-doped TiN coating under both test conditions. The greater surface roughness of the TiN:Ag coating, confirmed by the topographic analysis and roughness parameters (e.g., higher Sa, Spk, Svk values), likely contributed to the increased contact angle. According to the Wenzel and Cassie–Baxter models, surface roughness can enhance both hydrophilic and hydrophobic behaviour, depending on the intrinsic wettability of the material. In the case of TiN:Ag, the more developed surface structure appears to reinforce its hydrophobic tendency [30]. Both coatings exhibited hydrophilic behaviour, with contact angle values below 90°, a range generally considered favourable for osteoblast adhesion and proliferation. Previous studies have shown that moderately hydrophilic surfaces—characterised by contact angles within this range—facilitate protein adsorption, promote osteoblast differentiation, and accelerate early osseointegration processes [31,32,33].

### 3.3. Tribological Properties and Assessment of Surface Geometric Structure After Tribological Tests

**Figure 6:**
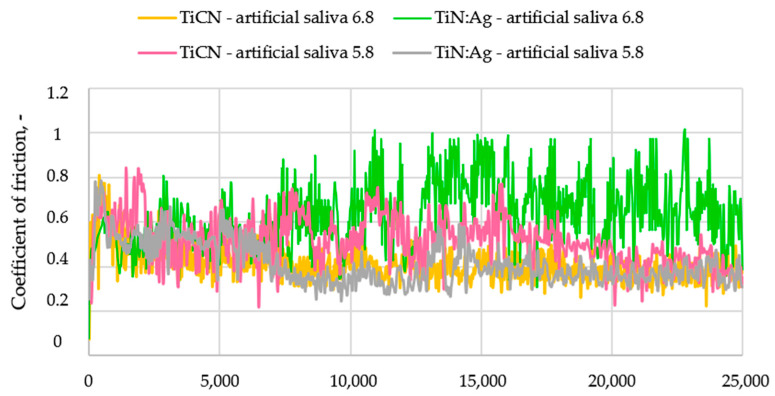
Examples waveforms of friction coefficients for TiCN and TiN:Ag coatings lubricated with artificial saliva at pH 5.8 and 6.8.

**Figure 7:**
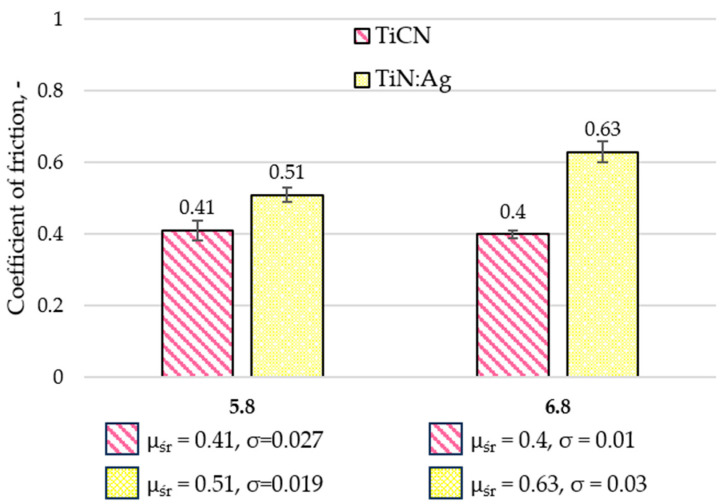
Average values of friction coefficients for TiCN and TiN:Ag coatings lubricated with artificial saliva at pH 5.8 and 6.8.

**Figure 8:**
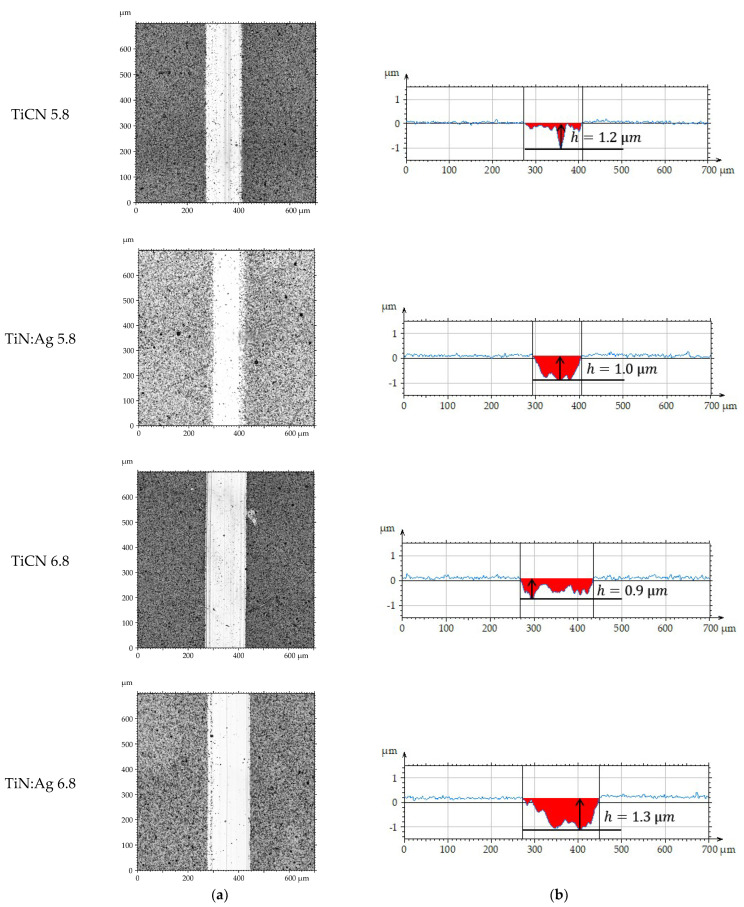
Views of wear track (**a**) and primary profiles on the cross-section (**b**) of TiCN and TiN:Ag coatings.

**Figure 9:**
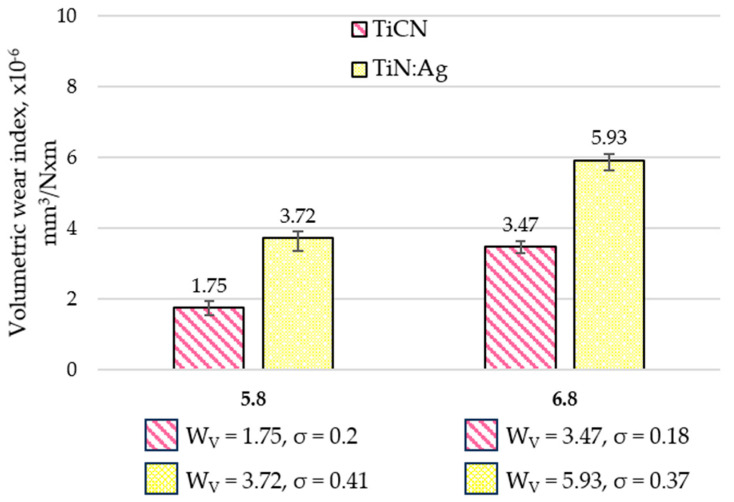
Average volumetric wear index values of TiCN and TiN:Ag coatings under lubrication with artificial saliva at pH 5.8 and 6.8.

Tribological tests were performed to determine the coefficient of friction (µ) and the wear intensity index (WV). Representative friction coefficient curves are shown in Figure 6, and the average values calculated from three measurement series are presented in Figure 7. Following the tests, the wear tracks were analysed using confocal microscopy. Based on the 3D axonometric images (Figure 8a), average wear profiles were generated (Figure 8b), and the cross-sectional wear area and wear depth (h) were determined. Based on the results of these tests, the volumetric wear index (Figure 9) was calculated using formula [34]: W_V_ = V/(F_N_ × S), where V-volume of material removed, mm^3^; F_N_–normal force, N; S–distance, m. These measurements enabled a quantitative evaluation of material loss and provided a basis for the comparative analysis of wear mechanisms operating on the examined coatings. Differences in the morphology and depth of the wear tracks offered insights into the dominant frictional interactions and the processes responsible for material degradation.

Tribological tests demonstrated that the TiCN coating exhibited lower coefficients of friction compared with the TiN:Ag coating under lubrication with artificial saliva at both pH 5.8 and 6.8. The measured friction values were approximately 20% and 36% lower, respectively. These differences are most likely associated with the higher surface roughness of the Ag-doped TiN coating. The reduced friction observed for the TiCN coating may result from the combined effect of two factors: the lubricating action of carbon present in the coating structure and the favourable mechanical properties of TiCN, including its high elastic modulus. The mechanical characteristics of both coatings, such as hardness and elastic modulus, have been comprehensively analysed in previous studies [35], which confirmed the superior mechanical performance of TiCN. Additionally, all tested samples exhibited a markedly unstable coefficient of friction throughout the measurement. This behaviour may be attributed to local electrochemical interactions between ions in the artificial saliva and the coating surface, as well as to differences in surface roughness between the examined materials.

Microscopic examination of the sample surfaces after tribological testing revealed that the TiCN coating exhibited significantly higher wear resistance than the TiN:Ag coating. This trend was consistent under both lubrication conditions, using artificial saliva at pH 5.8 and 6.8. No delamination or complete detachment of the coating from the substrate was observed in any of the analysed cases, as confirmed by the wear track profiles (Figure 8b). The depth of the wear tracks did not exceed the coating thickness, indicating that both layers remained intact throughout the tribological tests. According to previously reported data [35], the TiCN coating thickness was approximately 2.5 µm, whereas the TiN:Ag coating reached about 4.5 µm. These results further confirm the strong adhesion of both coatings to the Ti6Al4V substrate and their high resistance to tribological wear. Such characteristics are essential for applications requiring long-term durability and stability, particularly in dental implant components.

The volumetric wear index of the TiCN coating was approximately 52% and 41% lower than that of the TiN:Ag coating under lubrication with artificial saliva at pH 5.8 and 6.8, respectively. Both coatings exhibited more pronounced wear in the environment with pH 6.8—by about 50% and 37%, respectively—indicating the influence of the chemical composition of the lubricating medium on wear behaviour. 

The superior tribological performance of the TiCN coating can be attributed to the presence of carbon in its structure, which provides self-lubricating properties and contributes to reduced friction and wear. Furthermore, the high hardness and elastic modulus of TiCN [35] enhance its resistance to deformation and surface damage. The combination of these effects results in improved durability under cyclic loading conditions.

These results confirm that TiCN coatings exhibit strong potential for dental implant applications, where high wear resistance and stable frictional behaviour are essential for long-term functionality and clinical reliability.

## 4. Conclusions

The conducted analyses demonstrated clear differences in surface morphology, wettability, and tribological behaviour between the TiCN and TiN:Ag coatings. The TiN:Ag coating exhibited a more developed and irregular topography, characterised by higher roughness parameters and the presence of sharp surface asperities, which contributed to increased friction during the initial stages of sliding. In contrast, the smoother TiCN coating showed lower coefficients of friction under lubrication with artificial saliva at both pH 5.8 and 6.8—by approximately 20% and 36%, respectively. The superior tribological performance of the TiCN coating is likely associated with the presence of carbon within its structure, which provides additional self-lubricating effects. Wettability measurements confirmed that TiCN was more hydrophilic than TiN:Ag, a result consistent with its smoother surface morphology. Both coatings exhibited increased wear in the neutral saliva environment (pH 6.8), indicating that the chemical composition and pH of the lubricating medium influence the prevailing wear mechanisms. For TiCN, wear increased by about 50%, while for TiN:Ag it rose by approximately 37%. Microscopic examination further revealed that TiCN displayed superior wear resistance, with a volumetric wear rate up to 52% lower than that of TiN:Ag. These findings highlight the advantageous properties of the TiCN coating for dental implant applications. Its combination of high wear resistance, favourable frictional stability, and hydrophilic surface behaviour suggests strong potential for improved long-term functionality and clinical reliability under conditions simulating the oral environment.

## Figures and Tables

**Table 1:** Characteristics of the materials used for testing.

Coatings	Manufacturer	Deposition Technique	View
TiCN	Oerlikon Balzers, Montcada i Reixac, Spain	s3p	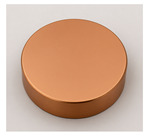
TiN:Ag	Oerlikon Balzers, Brügg bei Biel, Switzerland	arc	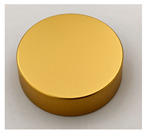

**Table 2:** Characteristics of the testing methods.

Methodology and Section Number	Measurement Instruments	Research Methodology
3.1. Microstructure and surface texture before tribological test	Phenom XL (Thermo Fisher Scientific, Eindhoven, The Netherlands) DCM8 non-contact profilometer (Leica, Heerbrugg, Switzerland)	sample shape: disc, Ø 18 mm, method: SED magnification: ×5000 sample shape: disc, Ø 18 mm, method and lens: confocal, ×20 measurement area: 0.65 mm × 0.85 mm
3.2. Wettability	Attension Theta Flex (Biolin Scientific, Tietäjäntie, Finland)	method: sessile drop method, liquid: artificial saliva 5.8 and 6.8 drop volume: 5 µL
3.3. Tribological Properties and Assessment of Surface Geometric Structure After Tribological Tests	TRB^3^ tribometer (Anton Paar, Baden, Switzerland)	sample shape: disc, Ø 18 mm, motion: reciprocating, load and cycle: 1 N, 25,000 amplitude and frequency: 10 mm, 1 Hz countersample: Al_2_O_3_ ball, diameter 6 mm lubrication: artificial saliva 5.8 and 6.8
	DCM8 non-contact profilometer (Leica, Heerbrugg, Switzerland)	sample shape: disc, Ø 18 mm, method and lens: confocal, ×20 measurement area: 0.7 mm × 0.7 mm

**Table 3:** Amplitude parameters.

	Sa	Sq	Sv	Sp	Ssk	Sku
	µm				-	
TiCN	0.1	0.2	2.1	2.6	2.4	25.7
TiN:Ag	0.2	0.3	2.9	8.6	3.7	56.9

**Table 4:** Parameters from the areal material ratio curve.

	Sk	Spk	Svk
	µm		
TiCN	0.38	0.34	0.18
TiN:Ag	0.39	0.45	0.21

## Data Availability

The original contributions presented in the study are included in the article, further inquiries can be directed to the corresponding author.
